# Surviving the Unthinkable: A Case of Post-obstructive Pulmonary Edema and Cerebral Edema in a Suicidal Hanging Survivor

**DOI:** 10.7759/cureus.70310

**Published:** 2024-09-27

**Authors:** Avinash Parepalli, Harshitha Reddy, Tushar Sontakke, Sourya Acharya, Neha Rahul

**Affiliations:** 1 Department of Medicine, Jawaharlal Nehru Medical College, Datta Meghe Institute of Higher Education and Research, Wardha, IND; 2 Department of Radiation Oncology, Jawaharlal Nehru Medical College, Datta Meghe Institute of Higher Education and Research, Wardha, IND

**Keywords:** airway obstruction, cerebral edema, hanging, hypoxia, pulmonary edema

## Abstract

Hanging, ligature strangulation, and manual methods can all result in fatal injuries such as fractures, dislocations, and brain swelling. This case involves a 30-year-old woman who survived a hanging incident but subsequently developed pulmonary and brain swelling. Post-obstructive pulmonary edema (POPE) is characterized by sudden onset non-neurogenic, non-cardiogenic fluid accumulation in the lungs following the removal of an acute upper respiratory tract obstruction. This report explores the patient's clinical presentation, disease progression, management strategies, and potential complications that clinicians may encounter during treatment, thereby enriching their understanding of possible outcomes and challenges in patient care.

## Introduction

Strangulation, resulting from pressure applied to the neck, can lead to suffocation. Hanging, ligature strangulation, and manual techniques are three common and medically significant forms of pressure [1]. Hanging, often chosen as a method of suicide due to its gravity-induced effects, can quickly lead to death. Near-hanging incidents occur when individuals are promptly rescued. The fatality associated with hanging can be attributed to fractures, dislocations, and brain swelling caused by ligature pressure, carotid artery obstruction, or cerebral ischemia. In severe cases, outcomes such as cerebral infarction, hypoxic-ischemic encephalopathy, acute respiratory distress syndrome, and inflammatory responses can result in swelling and death [1]. Post-obstructive pulmonary edema (POPE) is a sudden onset, non-neurogenic, non-cardiogenic fluid accumulation in the lungs following the rapid removal of an acute upper respiratory tract obstruction. It often manifests in the postoperative period after incidents like laryngospasm during intubation or anesthesia [1-2].

POPE is classified into two types: the first is triggered by forceful inhalation, acute and severe upper airway obstructions, as well as drowning, while the second arises when prolonged incomplete obstruction of the respiratory tract is relieved, such as in cases of hypertrophied adenoids or tonsils [1-3]. The specific mechanism behind the development of pulmonary edema in delayed hanging fatalities is not fully understood. Some theories suggest that disruptions in the pulmonary capillary membrane lead to pulmonary edema, hyperemia, and increased venous return. In contrast, others propose the release of vasoactive mediators, resulting in effects like congestion and cerebral hypoxia [1-2]. Compression, vascular damage, and hanging can all cause cerebral edema and hypoxic brain injury. Compression forces can injure the jugular veins and carotid arteries, leading to severe hypoxia and eventual death. Suicidal hanging typically results in less damage to the cervical spine [4]. Cerebral edema occurs due to cellular damage, leakage from blood vessels, and limited absorption pathways. It arises from a series of injuries, including glutamate release, influx of calcium and sodium, and hypoxia [5]. This results in intracellular accumulation of salts, water movement, calcium buildup, and inflammation. Cerebral edema can manifest as either vasogenic or cytotoxic. Vasogenic edema occurs when fluids breach the blood-brain barrier and damage white matter. At the same time, cytotoxic edema is caused by toxic substances released by neutrophils and bacteria that harm grey matter [5]. We present a case of a survivor of partial hanging who developed hydrostatic pulmonary edema and cerebral edema. This represents a rare illustration of the pathophysiological events that may occur in cases of airway obstruction. Our report aims to inform clinicians and researchers about the potential outcomes to be vigilant for in such scenarios.

## Case presentation

**Figure 1:**
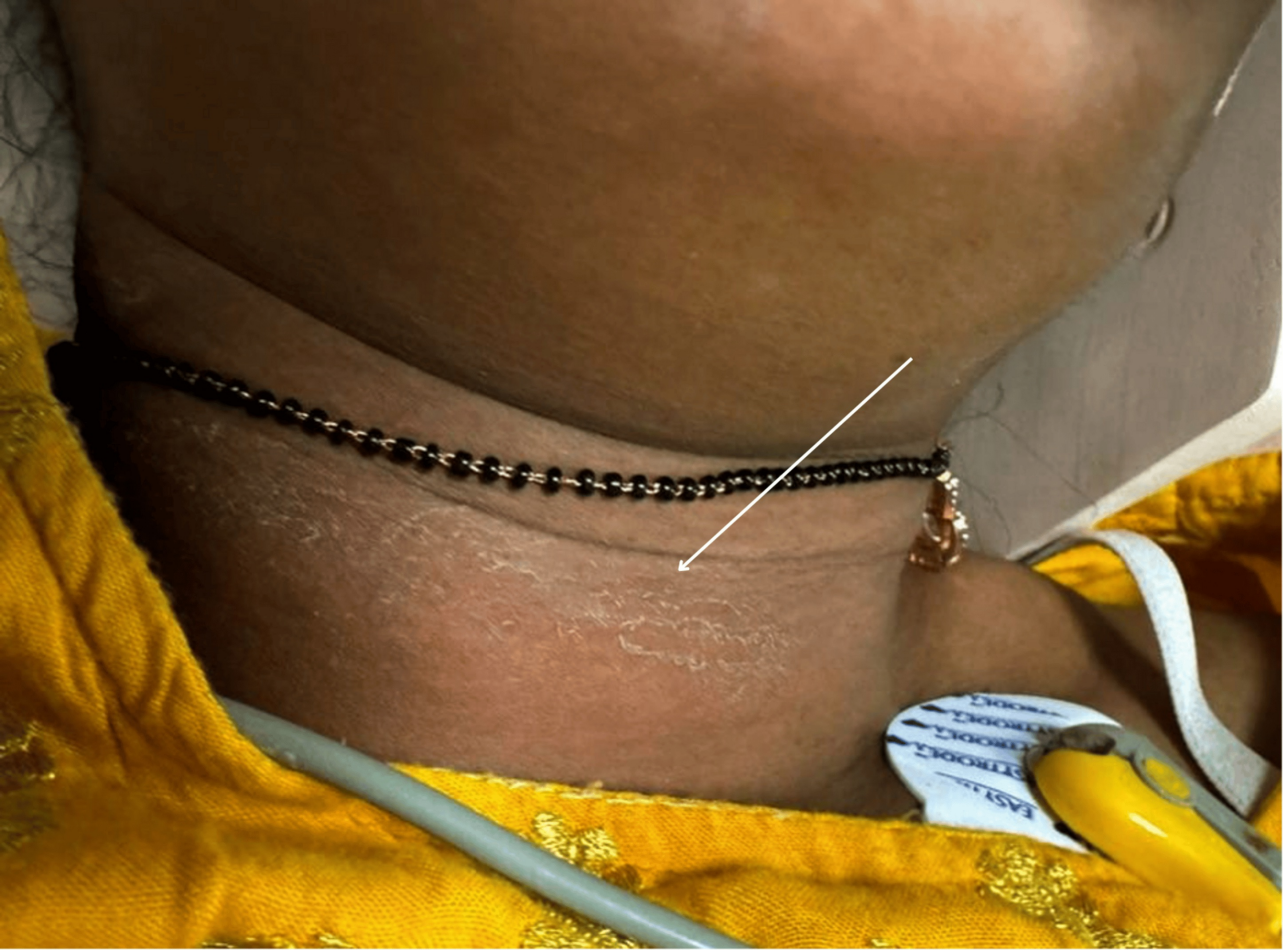
Partial ligature mark on the neck of the patient (white arrow)

A 30-year-old woman was brought unconscious to the emergency room after an incident of alleged self-harm by hanging at home using a saree for approximately three minutes. Her husband intervened by removing the saree from her neck and quickly lowering her to the ground. Despite his efforts to revive her, she remained unresponsive. The patient arrived at the hospital within 60 minutes. On assessment, she presented with a Glasgow Coma Scale (GCS) score of E1V1M1 (3/15), indicating severe impairment, and displayed rapid breathing. A partial ligature mark was visible around her neck (Figure 1).

**Figure 2:**
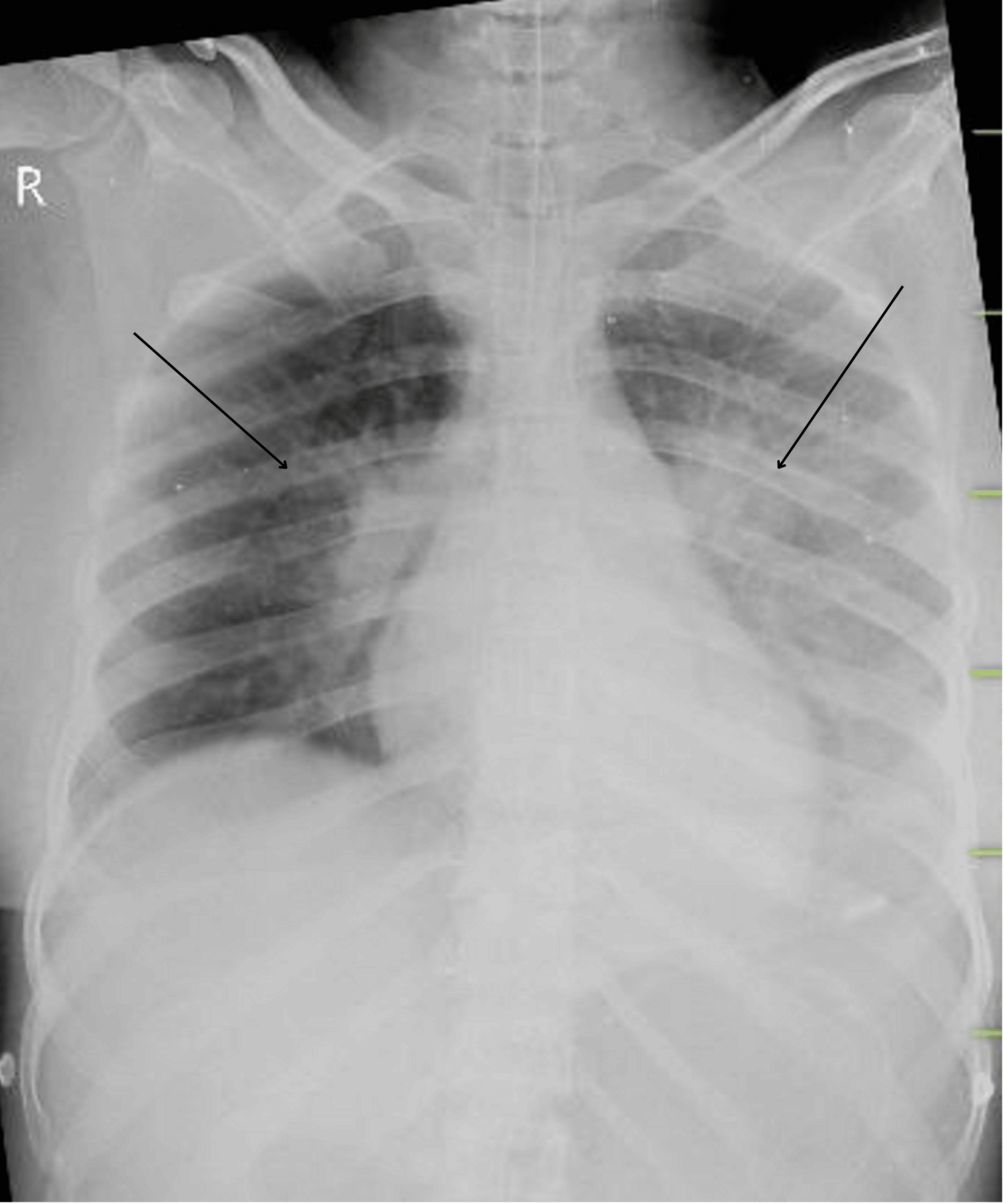
Chest radiograph of the patient showing hydrostatic pulmonary edema on the day of admission (black arrows)

Upon examination, the patient presented with a heart rate of 128 beats per minute, a blood pressure of 80/50 mmHg, a respiratory rate of 46 breaths per minute, and an oxygen saturation of 82% on pulse oximetry. Given her low GCS score, elevated heart rate, and increased respiratory rate, the medical team opted to perform endotracheal intubation and initiate mechanical ventilation for respiratory support. A rapid arterial blood gas (ABG) analysis revealed severe metabolic acidosis, with a pH of 7.012, partial pressure of carbon dioxide (pCO2) of 28 mmHg, partial pressure of oxygen (pO2) of 98 mmHg, oxygen saturation (SO2) of 95%, and bicarbonate (HCO3) of 16. Immediate correction of the metabolic acidosis was started using HCO3. A rigid cervical collar was applied to stabilize her neck and prevent further injury. Central venous access was established via the femoral vein to facilitate drug administration and monitoring. Pink, frothy secretions were observed in the endotracheal tube, indicative of pulmonary edema. An electrocardiogram (ECG) showed sinus tachycardia without ST segment abnormalities, reflecting an elevated heart rate. A chest X-ray revealed bilateral hilar prominence with a bat-wing appearance on the right side, consistent with hydrostatic pulmonary edema (Figure 2).

**Figure 3:**
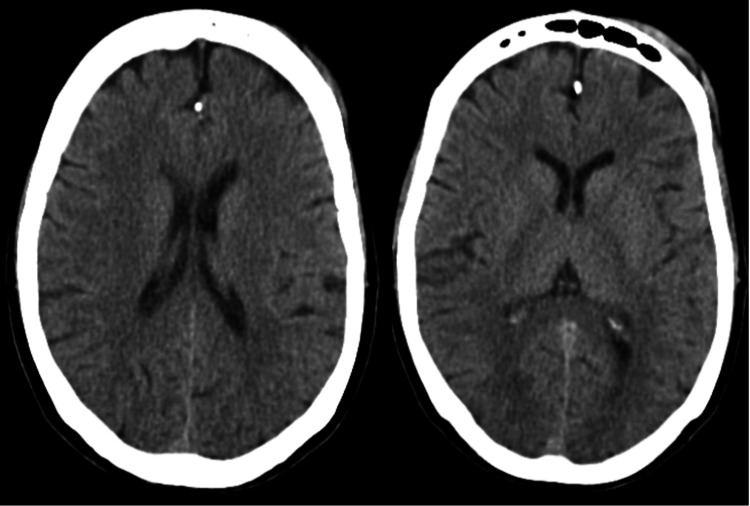
Computerized tomography of brain axial sections on the day of admission

A 2D echocardiogram was conducted to evaluate for underlying cardiac issues. The results indicated normal chamber size and ejection fraction (EF), with no evidence of valvular abnormalities, effectively ruling out acute myocarditis as a cause of pulmonary edema. Furthermore, computerized tomography (CT) scans of the brain and cervical spine performed upon admission did not reveal any significant findings (Figure 3).

Laboratory tests were conducted to assess the patient's condition better. The results of her laboratory parameters are detailed in (Table 1).

**Table 1:** Laboratory parameters of the patient

Lab parameters	Observed value	Normal range
Haemoglobin	11.7 gm%	13-17 gm%
Mean corpuscular volume	66 fL	83-101 fL
Total leucocytic count	23900 cells/cu mm	4000 – 10000 cells/cu mm
Neutrophils	85%	40-60%
Lymphocytes	10%	20-40%
Monocytes	04%	2-8%
Basophils	00%	0.5-1%
Eosinophils	01%	1-4%
Platelets	4.92 lakhs/cu mm	1.5–4.1 lakhs/cu mm
Urea	33 mg/dL	19-43 mg/dL
Creatinine	0.8 mg/dL	0.66-1.25 mg/dL
Sodium	140 mmol/L	137-145 mmol/L
Potassium	4.0 mmol/L	3.5–5.1 mmol/L
Calcium	7.9 mg/dL	8.4-10.2 mg/dl
Magnesium	2.2 mg/dL	1.6-2.3mg/dL
Phosphorous	2.5 mg/dL	2.5-4.5 mg/dL
Uric acid	1.4 mg/dL	3.5-8.5 mg/dL
Alkaline phosphatase	82 U/L	38-126 U/L
Alanine transaminase	36 U/L	< 50 U/L
Aspartate transaminase	33 U/L	17-59U/L
Albumin	4.0 g/dL	3.5-5 g/dL
Total bilirubin	0.7 mg/dl	0.2-1.3 mg/dl
Conjugated bilirubin	0.2 mg/dl	0.0-0.3 mg/dl
Unconjugated bilirubin	0.5 mg/dl	0.0-1.1 mg/dl
Random blood sugar	107 g/dL	90-140 g/dL
Lactate dehydrogenase	389 U/L	140-280 U/L
Thyroid-stimulating hormone	4.48 μIU/ML	0.465-4.68 μIU/ML
Free T3	4.01 pg/ml	2.77-5.27 pg/ml
Free T4	1.68 ng/dl	0.78-2.19 ng/dl
High-sensitivity C-reactive protein	102.20 mg/L	1.0 to 3.0 mg/L

**Figure 4:**
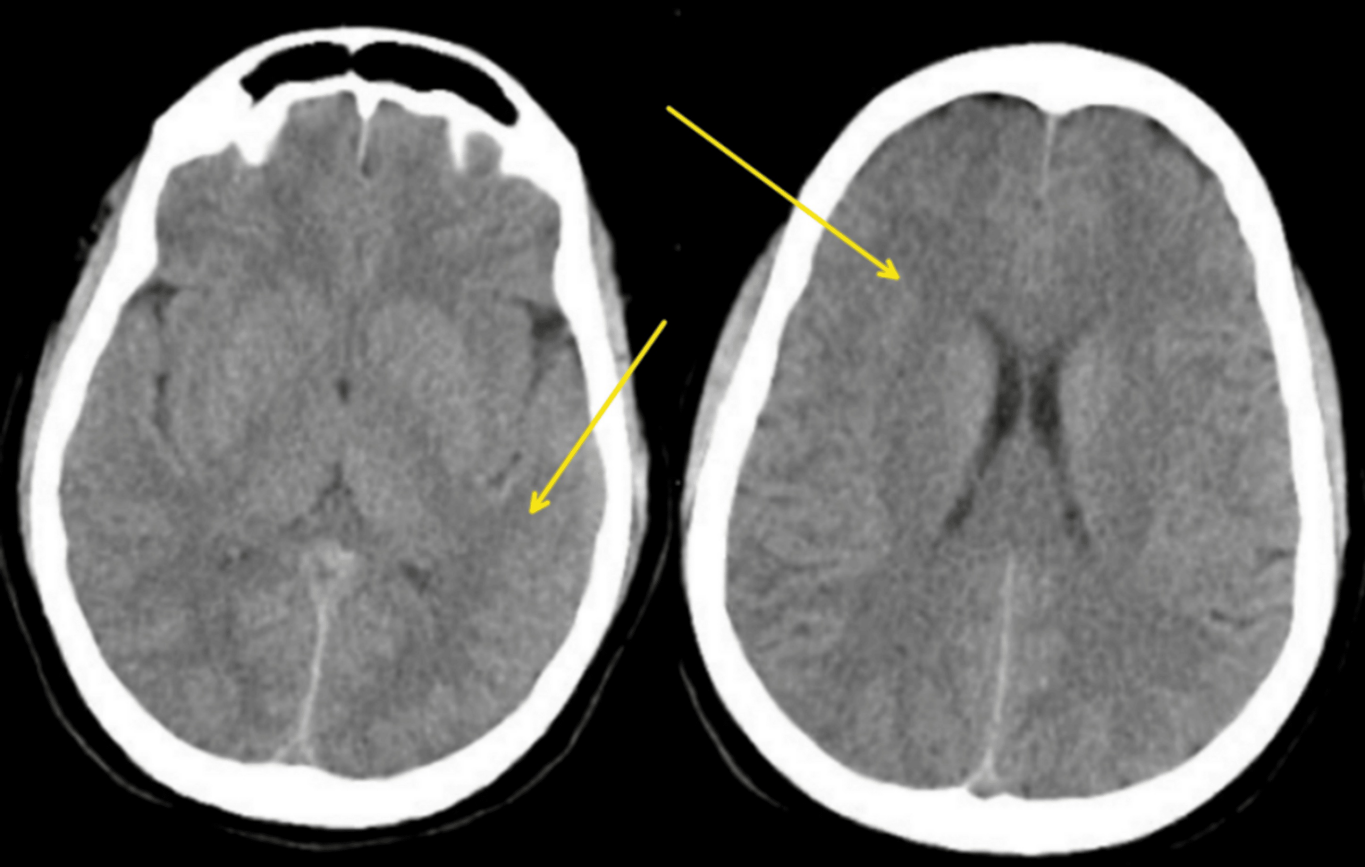
Computerized tomography of brain shows the cerebral edema on the second day (yellow arrows)

In response to hydrostatic pulmonary edema, the patient was started on a continuous infusion of injectable furosemide at 10 mg per hour to induce diuresis and reduce fluid accumulation in the lungs. Additionally, a single dose of 1800 mg methylprednisolone was administered to address inflammation and potential underlying causes of pulmonary edema. Following this, a maintenance infusion of methylprednisolone was prescribed to sustain its anti-inflammatory effects and support respiratory function. These pharmacological measures aim to alleviate pulmonary congestion and improve oxygenation. Close monitoring of the patient's response to treatment and adjustment of therapy as needed were crucial aspects of the intensive care unit management plan. By the second day of treatment, the patient's GCS improved to E4VTM6, and her vital signs stabilized: heart rate - 54/min, blood pressure - 160/70 mmHg, respiratory rate - 38/min, oxygen saturation on pulse oximetry - 98% on 40% fraction of inspired oxygen (FiO2). She remained on mechanical ventilation with the following settings: tidal volume - 360 ml, I ratio - 1:2, FiO2 - 40%, positive end-expiratory pressure (PEEP) - 6 cm H2O. Sedation was maintained, and the continuous furosemide infusion was stopped, transitioning to 40 mg of injectable furosemide intravenously twice daily. However, despite these interventions, the patient's level of consciousness did not improve as anticipated. A repeat CT scan of the brain was conducted, revealing signs indicative of extensive cerebral edema, including loss of normal grey-white matter differentiation, flattened or compressed sulci and gyri, compression of the ventricular system increased density of brain tissue (Figure 4).

**Figure 5:**
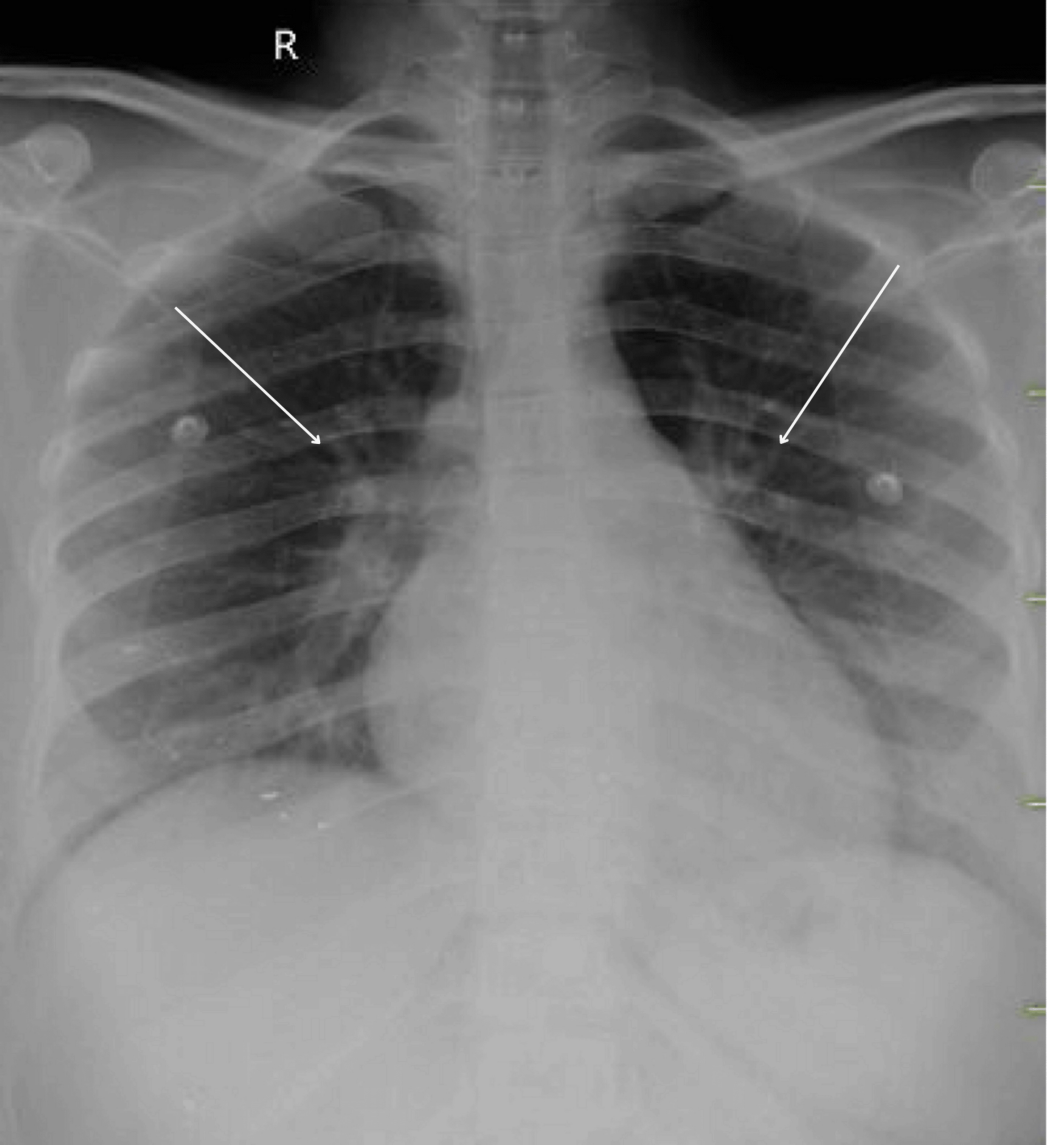
Chest radiograph of the patient showing decrease in the pulmonary edema (white arrows)

The patient began treatment with intravenous mannitol, administered as a single 300 ml dose initially, followed by 100 ml thrice daily, alongside intravenous levetiracetam at 500 mg twice daily. These interventions improved the patient's condition, leading to her extubation. She was subsequently transitioned to an oxygen mask, delivering 2 liters of oxygen. Daily chest radiographs thereafter indicated a gradual reduction in pulmonary edema, reflecting decreased fluid accumulation in the lungs. This positive imaging trend suggests a favorable response to therapeutic strategies to address the underlying causes of pulmonary edema. The diminishing pulmonary edema observed on chest radiographs underscores the effectiveness of diuretic therapy, oxygen supplementation, and other supportive measures in reducing excess lung fluid and improving respiratory function. Regular monitoring of pulmonary edema clearance through serial chest radiographs is crucial for evaluating the patient's clinical trajectory, guiding ongoing treatment decisions, and assessing the need for further interventions to optimize respiratory function and overall patient outcomes. On day 3, a chest radiograph revealed minimal interstitial thickening in bilateral lung fields with features indicative of reduced pulmonary edema (Figure 5).

**Figure 6:**
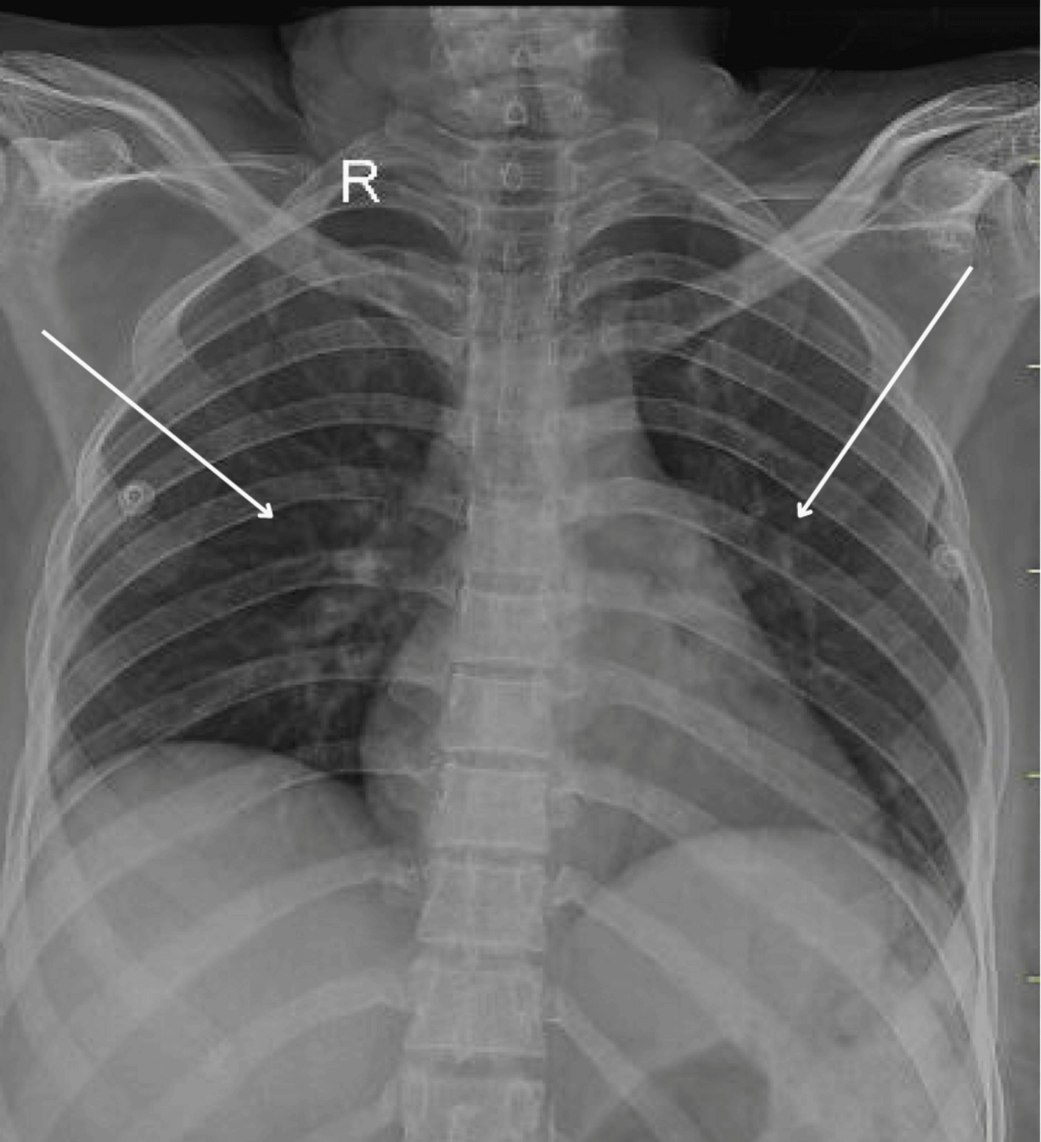
Chest radiograph with clear lung fields indicating the resolved pulmonary edema (white arrows)

On day 4, a chest radiograph indicated resolved pulmonary edema with no pleuroparenchymal abnormalities present (Figure 6).

**Figure 7:**
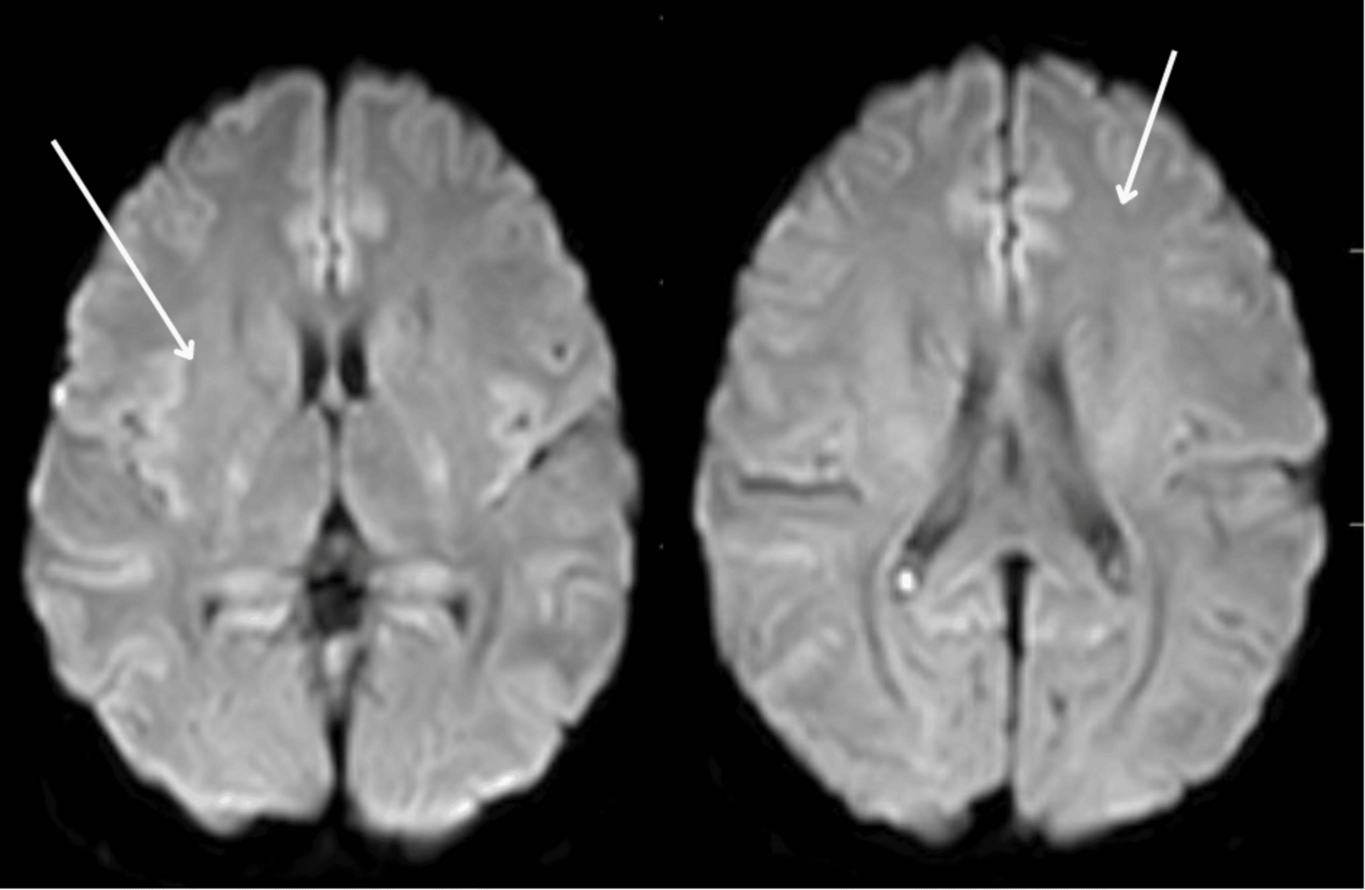
Magnetic resonance image of brain (diffusion-weighted image) with normal grey white differentiation in the brain parenchyma indicating the resolved cerebral edema (white arrows)

The patient underwent intravenous mannitol therapy for seven days, with close monitoring of hydration levels to ensure proper balance. Additionally, a magnetic resonance imaging (MRI) of the brain was performed, which showed a few nonspecific areas of T2/FLAIR (fluid-attenuated inversion recovery) hyperintensity in the right centrum semiovale and left frontal region. The brain parenchyma displayed normal grey-white matter differentiation (Figure 7).

The patient's treatment regimen, which included injectable mannitol and diuretics, was ceased, and symptomatic management was continued. The patient was moved from the intensive care unit to a general ward. Symptomatic care was upheld, and the patient was discharged with instructions for regular follow-up appointments.

## Discussion

Strangulation accounts for approximately 2.5% of all traumatic fatalities worldwide. Hanging, defined as the application of pressure to a person's neck until they are fully suspended, is a prevalent method of suicide both domestically and globally [6]. Hanging can lead to pulmonary complications such as aspiration pneumonia, acute respiratory distress syndrome (ARDS), and pulmonary edema due to increased negative intrathoracic pressure. Vascular occlusion, cerebral hypoxia, laryngeal swelling, carotid sinus stimulation, local trauma, and pulmonary issues collectively contribute to morbidity and mortality [6,7]. Typically, hanging fatalities result from spinal compression, cerebral ischemia, and cardiac failure due to compression of the airway and neck vasculature. Compression of the neck, airway obstruction, or mechanical asphyxia can lead to posterior displacement of the tongue. Injuries to the spinal cord, dislocations of cervical vertebrae, and fractures are common in judicial hangings, cases of degenerative cervical vertebral disease, and obese individuals who unexpectedly fall.

Various complications, such as hypoxic or ischemic brain injury, intracranial infarction or hemorrhage, and secondary cerebral damage, have been documented in near-hanging patients. Survival in near-hanging incidents hinges on factors such as duration of suspension, prompt resuscitation, constriction force, height of fall, point of suspension on the neck, hanging, time until release of compression, and the material used for hanging [1-8]. In our case, because a soft material, a saree, was used as the ligature, no fractures of the larynx, hyoid bone, or soft tissue hemorrhages were identified. The patient was promptly rescued, limiting the hanging duration to three minutes. Her feet were in contact with a surface below, indicating a partial hanging, and she was transported to the hospital within 60 minutes of the incident. POPE, or transudative pulmonary edema, is characterized by elevated pulmonary capillary pressure and vascular resistance resulting from the redistribution of blood from systemic to pulmonary vasculature. This form of pulmonary edema is often associated with airway obstruction, which may be caused by capillary leak syndrome, shearing pressures from forceful inspiration against a closed airway, or hypoxia [9]. The etiology of POPE II remains unclear; however, Type I POPE typically manifests within an hour following an event, whereas POPE II may be delayed for up to six hours, as might have been the case with our patient who developed hydrostatic pulmonary edema [9-10].

Hypoxia induced by a cessation in venous return can lead to congestive symptoms and potentially fatal outcomes [11]. POPE represents a potentially lethal complication of upper airway obstruction, particularly in non-fatal hangings [6-11]. Complications following hanging may include subpleural petechial hemorrhages, pink frothy edema fluid, and hyperemia. Hypoxia triggers vasoconstriction and increased pulmonary vascular permeability, resulting in elevated catecholamine levels. During brain injury and ischemia, as observed in our patient, mediators such as glutamate, free fatty acids, and increased extracellular potassium contribute to cellular swelling and neuronal death [5-12]. Substances like histamine, arachidonic acid, and free radicals can also induce cerebral edema. These mediators may interact in a cascade, eliciting responses that could potentially be mitigated pharmacologically.

## Conclusions

In conclusion, post-obstructive pulmonary edema (POPE) presents as a condition characterized by airway obstruction, often accompanied by symptoms such as restlessness, rapid breathing (tachypnea), elevated heart rate (tachycardia), frothy pink sputum, crackling sounds in the lungs (rales), and worsening oxygen levels. The diagnosis is typically considered in otherwise healthy young individuals with normal heart function and signs of pulmonary edema. Treatment involves the administration of supplemental oxygen and respiratory support, often including intubation with low positive end-expiratory pressure. The role of diuretics in managing POPE remains uncertain; however, the overall prognosis is favorable, with most patients showing rapid improvement with appropriate treatment.
